# Diagrammatic approaches to RNA structures with trinucleotide repeats

**DOI:** 10.1016/j.bpj.2021.04.010

**Published:** 2021-04-19

**Authors:** Chi H. Mak, Ethan N.H. Phan

**Affiliations:** 1 Department of Chemistry, Center of Applied Mathematical Sciences and Department of Quantitative and Computational Biology, University of Southern California, Los Angeles, California; 2 Department of Chemistry, University of Southern California, Los Angeles, California

## Abstract

Trinucleotide repeat expansion disorders are associated with the overexpansion of (CNG) repeats on the genome. Messenger RNA transcripts of sequences with greater than 60–100 (CNG) tandem units have been implicated in trinucleotide repeat expansion disorder pathogenesis. In this work, we develop a diagrammatic theory to study the structural diversity of these (CNG)_n_ RNA sequences. Representing structural elements on the chain’s conformation by a set of graphs and employing elementary diagrammatic methods, we have formulated a renormalization procedure to re-sum these graphs and arrive at a closed-form expression for the ensemble partition function. With a simple approximation for the renormalization and applied to extended (CNG)_n_ sequences, this theory can comprehensively capture an infinite set of conformations with any number and any combination of duplexes, hairpins, multiway junctions, and quadruplexes. To quantify the diversity of different (CNG)_n_ ensembles, the analytical equations derived from the diagrammatic theory were solved numerically to derive equilibrium estimates for the secondary structural contents of the chains. The results suggest that the structural ensembles of (CNG)_n_ repeat sequence with n ∼60 are surprisingly diverse, and the distribution is sensitive to the ability of the N nucleotide to make noncanonical pairs and whether the (CNG)_n_ sequence can sustain stable quadruplexes. The results show how perturbations in the form of biases on the stabilities of the various structural motifs, duplexes, junctions, helices, and quadruplexes could affect the secondary structures of the chains and how these structures may switch when they are perturbed.

## Significance

Trinucleotide repeat expansion disorders are associated with the overexpansion of (CNG) repeats on the genome. Messenger RNA transcripts of sequences with critical length greater than 60–100 (CNG) tandem units have been implicated in trinucleotide repeat expansion disorder pathogenesis, though their structures remain poorly characterized. The conventional view has tacitly assumed that conformations with maximal C:G basepairing dominate at equilibrium, but here we demonstrate that (CNG) repeat sequences are characterized by diverse ensembles of structurally heterogeneous folds and with a large variance of secondary structural contents. These results were based on a diagrammatic approach to the ensemble partition function.

## Introduction

Diagrammatic approaches for classifying RNA structures have been used widely (1, 2, 3, 4, 5, 6, 7, 8, 9, 10, 11, 12). Graphs provide an elegant method for categorizing the many diverse conformational structures that can be adopted by RNA sequences and may be used to more easily recognize common topological features in RNA structures that are otherwise difficult to decipher from their two- or three-dimensional structures. Graphs also provide an alternate space within which RNA secondary structures can be understood (13,14), and they are the basis of the algorithms (15,16) behind some of the most widely used RNA secondary structure prediction tools (17, 18, 19). Graphs also help elucidate the rich connection between RNA structure and topology, enabling topological interpretations to be used for annotating RNA structures (20, 21, 22, 23, 24, 25).

In this work, we employ diagrammatic methods to compute the conformational diversity of trinucleotide repeat RNA sequences. In a family of neurological diseases known as trinucleotide repeats expansion disorders (TREDs) (26, 27, 28, 29, 30), the onset of illness is associated with the overexpansion of (CNG)_n_ repeats in the genome (29, 30, 31). Although most of these expanded repeats occur in noncoding regions and do not appear to translate to aberrant proteins (30,31), the messenger RNA transcripts of these overexpanded templates may interfere with cellular pathways, leading to cytotoxicity (32,33). At the same time, (CNG)_n_ expanded messenger RNA may also acquire unintended functions in the cell (34). Ascertaining the structures of these sequences is therefore necessary for understanding their functions.

**Figure 1:**
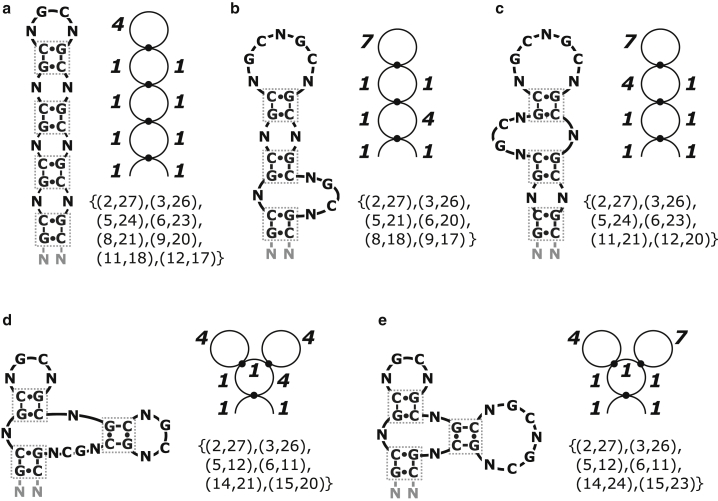
Examples of a 5′-NG(CNG)_8_CN-3′ repeat sequence in five different conformations. (*a*) The maximal hairpin “necklace” structure. (*b* and *c*) Structures with an asymmetric internal junction. (*d* and *e*) Structures with three-way junctions. The dual graph representation is shown next to each example, in which each 2-bp duplex is represented by a dot, hairpin loops are represented by circles with one dot, two-way junctions by circles with two dots, and three-way junctions by circles with three dots, and an arc represents the two unpaired ends. In the graphs, the number adjacent to each edge indicates its length in nt. The basepair representation is shown below the dual graph of each example.

Examples of some possible conformations of a short (CNG) repeat with different secondary structures are shown in Fig. 1. Because of their repeat structures, at least one-third of the nucleotides on (CNG)_n_ sequences cannot form canonical basepairs upon folding. Depending on the identity of the N nucleotide, they may also interact with themselves or with the G or C nucleotides. TRED disease onset is often associated with a critical expansion threshold of n > 60–100 (35). The structures most often associated with the gain-of-function hypothesis for CNG-expanded RNA sequences cited in the literature is a necklace-like structure composed of a long stretch of successive two-way junctions interposed by shorts helices and with a hairpin stem-loop cap (31,32,36, 37, 38, 39, 40), like the one shown in Fig. 1 *a*. Many of the studies conducted are based on short (CNG) repeats (31,32), and the structures resolved are limited to those that can be isolated and crystalized (38, 39, 40, 41). As the length of the CNG repeats grows, the diversity of accessible structures could grow rapidly as well.

Fig. 1*a* illustrates a maximally canonically paired “necklace” structure. To the right of it is shown its dual graph representation. The length of each junction is specified in number of nucleotides (nt). The basepair representation of the structure is shown below the dual graph. The basepair or “matrix” representation explicitly enumerates the sequence positions of the nucleotides bound by canonical interactions. Fig. 1, *b* and *c* show two other examples in which one of the two-way junctions is asymmetric. These two structures have one fewer helix and thus lower basepair and stacking stability than Fig. 1 *a*. Their dual graph representations are shown to the right of Fig. 1, *b* and *c*, suggesting that their loop structures are topologically distinct from Fig. 1 *a*. Different junction lengths also cost different amount of conformational entropy for the sugar-phosphate backbone. The loop entropies in the various secondary structures must be accounted for to correctly determine their free energies. In general, Fig. 1, *b* and *c* do not have the same loop entropies even though they contain the same number of nucleotides inside their loops (five 1-nt loops, one 4-nt loop, and one 7-nt loop). This is because the 4 × 1 internal loops in Fig. 1 *c* adjacent to the hairpin may sterically interface with each other and with the helices differently compared to the 1 × 1 internal loops in Fig. 1 *b*. Loop entropies are therefore dependent on where and how they appear on the structure relative to each other.

Fig. 1, *d* and *e* show two examples with three-way junctions. In general, higher multiway junctions cost more entropy because they represent a more stringent conformational constraint for the sugar-phosphate backbone, and they also experience more steric congestion for the helices around the junction. The dual graph representation of each is shown to the right. Even though Fig. 1, *d* and *e* are topologically equivalent, they do not contain the same loop entropies because their loops are arranged differently along the sequence. Notice that although Fig. 1 *e* has identical junction lengths to Fig. 1, *b* and *c*, the loop entropies of these three structures are also intrinsically different.

Entropies of loops and junctions, or more precisely the loss in their conformational entropies, arise from constraints coming from the basepairs. An unfolded RNA is in a high-entropy state. Its structures are characterized by a diverse ensemble. If *c* denotes a chain conformation and *P*(*c*) its probability, the total entropy content of this ensemble is given by *S* = −*k*_*B*_$\sum_{c}$*P*(*c*)ln*P(c)*. If the sequence spontaneously folds and develops secondary and/or tertiary structures, the conformational entropy of the chain is suppressed because base complementarity and stacking interactions produce constraints on the chain’s conformations. Under these constraints, the new probability for each conformation in the presence of these constraints *P*′(*c*) = *P*(*c*|constraints) incurs a penalty, and the loss of entropy is given by

(1) $$\text{Δ}S=S\left(\text{with}\;\text{constraints}\right)-S\left(\text{no}\;\text{constraints}\right)=-k_{B}\sum_{c}P_{c}^{\prime }\;\ln\;P_{c}^{\prime }-P_{c}\;\ln\;P_{c},$$

where the sum runs over all conformations. If one can determine how the constraints imposed by the secondary and tertiary structures in the fold transforms *P*(*c*) → *P*′(*c*), Δ*S* can be determined.

In general, the constraints imposed by secondary or tertiary structures are correlated. “Factorizability” describes how these constraints may break up into independent (or approximately independent) subsets. For instance, if the fold introduces four constraints *A*, *B*, *C*, and *D* but the effects of *A* and *B* are separable from *C*, which is also separable from *D*, then *P*′(*c*) = *P*(*c*|*A*, *B*, *C*, *D*) = *P*(*c*|*A*, *B*) × *P*(*c*|*C*) × *P*(*c*|*D*). Under this factorization, the entropy change in Eq. 1 would simply be equal to Δ*S* = Δ*S*(with constraints *A*, *B*) + Δ*S*(with constraint *C*) + Δ*S*(with constraint *D*).

Different approximations have been used to account for loop entropies in RNA folding predictions. These range from ignoring loop entropies all together (20,23,42,43) to treating each loop in the secondary structure as independent and approximating its value by additivity rules (13, 14, 15) to assigning experimentally derived free energy to loops of specific known sequences (44). The most sophisticated of these is NNDB (45), which Mfold (17) is based on. NNDB employs thermodynamic data to assign approximate functional forms to interpolate experimentally measured loop free energies of hairpins, bulges, internal loops, and multibranch junctions. In one form or another, an intrinsic factorizability in the loop entropies is assumed by all of these approaches. For example, NNDB treats the loop entropies in multiway junctions higher than two approximated by a sum in the form *a* + *b* × *u* + *c* × *h*, where *u* is the number of unpaired nucleotides, *h* is the number of branching helices, and the empirical constants *a*, *b*, and *c* are parameters that were found by maximizing the accuracy of secondary structure prediction (46). For many RNA folding problems, this assumption may be well justified because the thermodynamic driving force for the secondary structure comes from the stability of the pairing and stacking of bases in the helices. But for (CNG) trinucleotide repeat sequences, this may not be the case because each helix is no more than a two-basepair stack of GC|CG, and they lack the more substantial stacking free energy that stabilizes longer helices (47). Indeed, experimental measurements suggest that the helix free energy estimated from Mfold greatly overestimates the stability of GC|CG stacks in (CNG) repeats (36). Because of this, the role of the loop entropies, their factorizability, and how they influence the conformational diversity of (CNG) repeats should be examined.

Using a large body of empirical data derived from Monte Carlo (MC) conformational sampling (48,49), we have determined cases in which constraints are approximately independent and provided quantitative metrics for their factorizability. For example, in a two-way junction, the loop entropies of the two junctions are correlated, but they are largely independent from the loops on the other sides of the helices. The same is true for hairpins and other multiway junctions. The topological reason behind this loop factorizability is related to the secondary nature of these features. Furthermore, (48,49) provide a self-consistent library of loop entropies derived from MC simulations. The data library in (48,49) has been used in this study to more accurately account for these loop entropy contributions in conformational predictions for (CNG) repeats. In Materials and methods, we show how this approximate factorizabilities of the loop entropies can be expressed diagrammatically, and in Results and discussion, we apply this to study the conformational diversity of (CNG) repeat sequences.

## Materials and methods

### Graph representations

Tinoco et al. (50) used an adjacency matrix representation to denote the canonically bound basepairs in RNA secondary structures. This representation is given in Fig. 1 to the lower right of each structure. Waterman et al. (13,14,51) have described several equivalent representations, such as chord diagrams and linear trees. Schlick et al. (1,5,9) employed dual graphs to represent the same information, and examples of these are shown in Fig. 1 to the upper right of each structure. Though topologically equivalent, various representations emphasize different aspects of the folding free energies. The matrix representation and the chord diagrams, for example, emphasize the paired bases, whereas dual graphs highlight the unpaired segments on the loops and junctions, as pointed out by Liu and Bundschuh (44).

**Figure 2:**
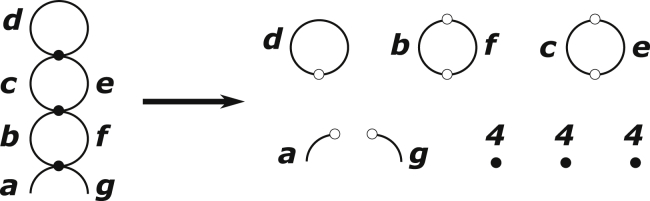
Example showing factorization of the diagram on the left into the factors on the right. The circle with one dot represents a hairpin loop of size *d*. Circles with two dots represent two-way junctions. The two open line segments represent open strands. The three filled dots represent 2-bp (4-nt) duplexes. The corresponding expression for the composite probability is given in Eq. 2.

Because the focus of this work is on loops, we rely on dual graphs. In the Introduction, we describe the approximate factorizabilities of certain secondary structural features that were observed in the MC data in (48,49). These factorizabilities can be expressed using diagrams. For instance, the loop entropies of the unpaired segments in any two-way junction are correlated, but they are largely uncorrelated with the loops on the other sides of the helices exiting from the two-way junction. Fig. 2 shows how this factorization works for the two structures in Fig. 1, *b* and *c*. Each of the objects on the right side of Fig. 2 contain loop entropies that can be retrieved from the data library in (48,49). Similar factorizabilities exist for higher multiway junctions, and their dual graph representations can also be used to express this in the same way analogous to Fig. 2.

The composite probability of the graph on the left in Fig. 2 is given by

(2) $$P_{1}\left(d\right)P_{2}\left(b,f\right)P_{2}\left(c,e\right)P_{0}\left(a\right)P_{0}\left(g\right){\left[P_{\cdot }\left(4\right)\right]}^{3},$$

where *P*_1_(*x*) is the probability associated with a hairpin loop (or a “one-way junction”) of length *x*, *P*_2_(*x*, *y*) is the probability of a two-way junction with loop lengths *x* and *y*, *P*_0_(*x*) = 1 is the probability associated with an open strand, and *P*. is the probability of the duplex. For the loops in hairpin and junctions, their probabilities are given by $P=e^{\text{Δ}S/k_{B}}$, where Δ*S* is the conformational entropy of a loop relative to an open strand. *P*.(4) = $e^{\text{Δ}S_{\cdot }/k_{B}-\text{Δ}H_{\cdot }/k_{B}T}$, the probability of a 2-bp (4-nt) duplex, has both enthalpic and entropy contributions in it, which involve stacking and basepairing interactions as well as the loss of conformational freedom suffered by the backbone to stack. An example of all the decomposable factors of a necklace diagram is given on the right side of Fig. 2.

### Specializing to (CNG) repeat sequences

To specialize the formulation to apply to 5′-NG(CNG)_8_CN-3′ repeat sequences specifically, we take into account their repeat structure. By “repeat structure,” we are referring to the periodicity of the nucleotide sequence. In our calculations, we employ constructs with the following architecture:

$$5^{’}\text{-}\left(N\text{-}GC\right)\text{-}\left(N\text{-}GC\right)\text{-}\left(N\text{-}GC\right)\text{-}\left(N\text{-}GC\right)\text{-}\left(N\text{-}GC\right)\text{-}\left(N\text{-}GC\right)\text{-}\ldots \text{-}\left(N\text{-}GC\right)\text{-}\left(N\text{-}GC\right)\text{-}N\text{-}3^{’}$$

with *n* repeating units of (NGC). Formally, this construct has *l* = 3*n* + 1 nucleotides instead of 3*n*. This is done to ensure that the 5′ and 3′ ends of the chain do not have to be treated differently, but it does not materially alter the results or the formulation.

As described above, the periodicity of the sequence permits canonical basepairing producing 2-bp duplexes only. Beyond that, the ability of the N nucleotides to form noncanonical basepairs can favor different structures depending on whether N = A, C, G, or U. These noncanonical effects can be captured by assigning an extra bias to the two-way junctions of those sequences in which a noncanonical basepair or stacking can add stability to the chain. Because of the repeat structure, unpaired segments on the sequence are limited to lengths equal to 1, 4, 7, 11, … nt. To do this, every loop length in the formulation is replaced by its length divided by 3. For example, the lengths {*a*, *b*, … *g*} in Fig. 2 become {*a*′ = *a*∖3, *b*′ = *b*∖3, … *g*′ = *g*∖3}, where ∖ denotes an integer division without remainder. A loop with length *a*′ = 0 is 1 nt long. A loop with *a*′ = 1 is 4 nt long, etc. The only exception to this rule is a 2-bp (4-nt) duplex, which is assigned a length of two repeat units instead of 1, and a quadruplex, which is assigned four repeat units.

Bundschuh et al. (44,52) have applied a related diagrammatic method to various trinucleotide repeats. They employed a diagrammatic recursion relation for the partition function *Z* to study the crossover from asymptotic scaling behavior to finite-length effects. They found that in the presence of multiloop junctions, the crossover to the scaling regime is related to the chain’s ability to make branches. For (GCA)_n_ chains, their results show that the scaling regime is reached with just a handful of repeats, whereas for (GCC)_n_ sequences, the crossover does not occur until the sequence is hundreds of repeats long because of the extra pairing coming from the N = C nucleotides in the junctions with the G residues adjacent to them. These studies suggest that the interaction of the N nucleotide in (CNG) repeats may play a significant role in determining their prevalent structures. In our work, we have employed a graph renormalization scheme based on diagrammatic decomposition to study the concentrations of different structural elements on the chain, whereas in the work of Bundschuh et al. (44,52), their graph recursion on *Z* was better suited to studying the emergence of repeat-length-dependent asymptotic behaviors. But the two methods share common diagrammatic features.

### Graph elements and loop entropy contributions

**Figure 3:**
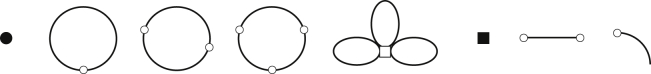
Dual graph representation of all structural elements included in this study: helix, hairpin, two-way junction, three-way junction, loops in a quadruplex, the quadruplex core, bridge, and unpaired ends.

The secondary structural elements considered in this study are shown in Fig. 3. A dot represents a GC|CG helix. Its probability *P*. contains the pairing and stacking free energy, as well as the backbone entropy of the doublet. Circles with one, two, or three holes represent the loops in a hairpin, a two-way junction, and a three-way junction, respectively, and their probabilities *P*_1_, *P*_2_, and *P*_3_ contain the loop entropies. Hairpins and two-way junctions have been found in experimental thermodynamic studies (36) to be most relevant for (CNG) repeat sequences. In this study, we also include three-way junctions to assess their relevance. In addition to these, quadruplexes, represented by the diagram with three loops emanating from a square core in Fig. 3, have also been included because they have been observed in experimental studies of other trinucleotide repeat sequences, noticeably (AGG) and (UCC) (36). The core of each quadruplex contains a double-deck tetrad structure with eight G nucleotides bound with Hoogsteen basepairs and is represented diagrammatically by a solid square. Its probability *P*_*q*_ contains the pairing and stacking free energy as well as the backbone entropy of the bases in the tetrad. Because only G can form tetrads, quadruplexes are possible only on the (CGG) repeat sequence. For multibranch structures, although we have limited ourselves to a three-way junction in this work, four-, five-, or any higher multiway junctions may be added without complications, but the results will show that multiway junctions are of less importance for (CNG) repeats. The 5′ or 3′ unpaired ends of the chain, represented by the last diagram in Fig. 3, do not cost any extra entropy compared to an open chain.

The loop entropies contained in each graph element are supplied by the data library in (48,49). For example, the entropies of the two loops in a two-way junction are dependent, but their total can be expressed as a function of the sum of their lengths. The portions of the library relevant to (CNG) repeats are reproduced in Table 1 for total loop length in units of the number of repeats *n*. Loop entropy data for all relevant elements in Fig. 3 are given in Table 1.

**Table 1:** Contributions of loop entropies to the folding free energy at 310 K

Feature (nickname)	Loop free energy as a function of total loop length (kcal/mol)				
	n = 0	n = 1	n = 2	n = 3	n > 3
Hairpin (1wj)	∞	5.02	5.85	6.16	3.9 + 1.08 ln(3n + 1)
Two-way junction (2wj)	5.97	6.53	6.79	6.88	4.4 + 1.08 ln(3*n* + 2)
Three-way junction (3wj)	7.12	7.33	7/46	7.53	4.9 + 1.08 ln(3*n* + 3)
Quadruplex (quad)	15.5	17.6	19.0	19.9	∞

Contributions taken from the data library in (48,49) (*RT* = 0.616 kcal/mol). Entropies of the loops in a multibranch junction are in general correlated, but their sum scales with the total junction lengths. Loop entropies of the junction internal to the branches are uncorrelated with the loops on the other sides of the branches. Empirically, higher multibranch structures cost more entropy.

The basic premise of this work considers free energies of the loops to be a fundamental determinant of RNA structures. This is somewhat different from the traditional view, in which basepairs in helices, triplexes, quadruplexes, or from tertiary interactions are considered the drivers. Both of these factors are, of course, present in any RNA system, but in some problems paired structures are more important, whereas in others, loop entropies may outweigh pairs. For the type of problem studied in this work, in which the ensemble may be dominated by open instead of strongly paired structures, careful consideration must be given to the loop entropies. Our results will show that for the (CNG) repeats, treating the loop entropies carefully is the key to understanding their conformational ensembles.

### Stabilities of GC|CG helix doublets and G-quadruplexes

The core thermodynamic stabilities of paired structures, such as the helices and quadruplexes in Fig. 3, are taken from experiments. For example, to determine the free energy contribution from each duplex, we used the experimental Δ*G*_exp_ data reported by Sobczak et al. for (CNG)_20_ oligomers in 100 mM NaCl (36) for N = A, C, G, and U. The only conformation that was reported for (CNG)_20_ has the maximal hairpin structure, analogous to that shown in Fig. 1 *a*. Using the loop entropy values from our library and in conjunction with the experimentally observed Δ*G*_exp_ for the maximal hairpin, we determined the free energies of the helix cores in each of the (CNG)_20_ repeats for N = A, C, G, and U separately. The smallest came from N = C with Δ*G*_0_(duplex) = −6.17 kcal/mol, followed by U (−6.39 kcal/mol), A (−6.57 kcal/mol), and G (−6.62 kcal/mol). In the results below, we will use the N = C Δ*G*_0_(duplex) value as the reference, as this represents a lower bound to stability. The other results for N = A, G, or U were obtained by applying the appropriate offset to the values for each duplex. For quadruplexes, experimental data from Sobczak et al. (36) suggest that (UGG)_17_ and (AGG)_17_ can form quadruplexes, but (CGG) repeats cannot. To estimate the effects of including quadruplexes in the (CNG)_n_ repeat ensembles, we used the experimental free energies of (UGG)_17_ and (AGG)_17_ and determined the free energy of a quadruplex core using the Δ*G*_exp_ for (UGG)_17_ and (AGG)_17_ in 100 mM NaCl (36) These yielded an approximation for the quadruplex core free energy ∼−20.4 kcal/mol from (AGG)_17_ and (UGG)_17_. In our calculations, we varied the quadruplex stability from zero up to and beyond these values to examine how the potential formation of quadruplexes might affect the structures of (CNG) repeats.

The values of the duplex free energies derived from the experimental data of Sobczak et al. (36) using the method above are ∼3 kcal/mol weaker per GC|CG helix compared to the nearest-neighbor model of Turner et al. (45,53). Using Mfold (17) to calculate the free energy of a typical CNG repeat produces exclusively the maximal hairpin structure analogous to Fig. 1 *a* as the only significant conformation. But, using the helix free energies obtained according to the prescription in the last paragraph, structural alternatives to the maximal hairpin become more competitive. In general, non-maximally paired structures enjoy higher entropies because loop segments in hairpins and junctions are less constrained compared to paired bases. In the results below, we will see the tradeoff between higher entropy in the more open structures versus the higher stability in the helices and quadruplexes in compact structures produces a mixed diverse ensemble for most (CNG) repeat sequences, rather than favoring a single dominant maximal hairpin structure.

### Diagrammatic renormalization

The graph approach described here shares many features with those employed in field theory and in liquids, in which diagrammatic techniques have been used extensively to manipulate graphs (54). Previous work has also applied diagrammatic techniques to study RNAs (13,15,20,23, 24, 25,44,52).

**Figure 4:**
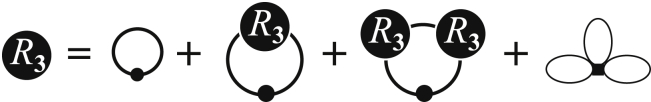
Dyson equation for the root function *R*_3_ including hairpins and two- and three-way junctions, as well as quadruplexes.

The canonical partition function of the ensemble *Z*(*n*) as a function of the number of (CNG) repeats *n* is represented by diagrams. The generating function, *Z*(*λ*) = $\sum_{n=0}^{\infty }$*Z*(*n*)exp(−*λn*), which is the grand canonical ensemble partition function allowing variable repeat lengths, can then be expressed in terms of the generating functions of the probabilities of the diagrammatic elements described above at 310 K. Standard renormalization allows the graphs to be re-summed, giving

(3) $$Z\left(\lambda \right)=1/\left[1-e^{-\lambda }-R\left(\lambda \right)\right],$$

where the root function *R* is a sum over all irreducible diagrams. Recursion relations similar to those in Eq. 3 have previously been described in the context of RNA structural studies (13, 14, 15,20,23, 24, 25,42,44,52). Pillsbury et al. reported similar recursion relations for RNA (42), as do Reidys et al. (43), and the use of irreducible diagrams has been introduced by Orland et al. (20,22,24,42) for studying RNA structures. The root function satisfies the Dyson equation (20,22,24,42,55), which is shown diagrammatically in Fig. 4. Including multibranch loops up to three-way junctions, this self-consistent equation for the root function *R*_3_(*λ*) is quadratic. Recursion relations for *Z* have also been used by Liu and Bundschuh (44) to examine how the partition function scales with repeat lengths.

The inputs *P*.(*λ*), *P*_1_(*λ*), *P*_2_(*λ*), *P*_3_(*λ*), and *P*_*q*_(*λ*) were obtained from the loop free energies of duplexes, hairpins, and two- and three-way junctions, as well as quadruplexes and the duplex and quadruplex stabilities described in the last subsection. The functional dependence of the loop free energies on the loop lengths were extended beyond the finite-length data available from the simulations by using the same scaling relationships that have been adopted by Turner et al. in the nearest-neighbor model (45,56,57), which was based on Stockmayer et al. (58), yielding the following expressions at *T* = 310 K:

(4a) $$P_{\cdot }\left(\lambda \right)=e^{-\left(2\lambda -\frac{6.17}{0.616}\right)},$$

(4b) $$P_{1}\left(\lambda \right)=e^{-\left(\lambda +\frac{5.016}{0.616}\right)}+e^{-\left(2\lambda +\frac{5.848}{0.616}\right)}+e^{-\left(3\lambda +\frac{6.159}{0.616}\right)}+e^{-\left(4\lambda +\frac{5.086}{0.616}\right)}\times \text{Φ}\left(e^{-\lambda },1.75,\frac{13}{3}\right),$$

(4c) $$P_{2}\left(\lambda \right)=Q_{2}\left(\lambda \right)-dQ_{2}\left(\lambda \right)/d\lambda ,$$

(4d) $$Q_{2}\left(\lambda \right)\equiv e^{-\left(\frac{5.970}{0.616}\right)}+e^{-\left(\lambda +\frac{6.528}{0.616}\right)}+e^{-\left(2\lambda +\frac{6.797}{0.616}\right)}+e^{-\left(3\lambda +\frac{6.880}{0.616}\right)}+e^{-\left(4\lambda +\frac{5.587}{0.616}\right)}\times \text{Φ}\left(e^{-\lambda },1.75,\frac{14}{3}\right),$$

(4e) $$P_{3}\left(\lambda \right)=\frac{1}{2}\left[2Q_{3}\left(\lambda \right)-3\frac{dQ_{3}\left(\lambda \right)}{d\lambda }+\frac{d^{2}Q_{3}\left(\lambda \right)}{d\lambda^{2}}\right],$$

(4f) $$Q_{3}\left(\lambda \right)\equiv e^{-\left(\frac{7.124}{0.616}\right)}+e^{-\left(\lambda +\frac{7.327}{0.616}\right)}+e^{-\left(2\lambda +\frac{7.458}{0.616}\right)}+e^{-\left(3\lambda +\frac{7.524}{0.616}\right)}+e^{-\left(4\lambda +\frac{6.087}{0.616}\right)}\times \Phi \left(e^{-\lambda },1.75,\frac{15}{3}\right),$$

and

(4g) $$P_{q}\left(\lambda \right)\equiv e^{-\left(4\lambda +\frac{20.4}{0.616}\right)}\left[e^{-\left(\frac{15.5}{0.616}\right)}+3e^{-\left(\lambda +\frac{17.6}{0.616}\right)}+3e^{-\left(2\lambda +\frac{19.0}{0.616}\right)}+e^{-\left(3\lambda +\frac{19.9}{0.616}\right)}\right],$$

where Φ is the Lerch transcendent (59).

## Results and discussion

**Figure 5:**
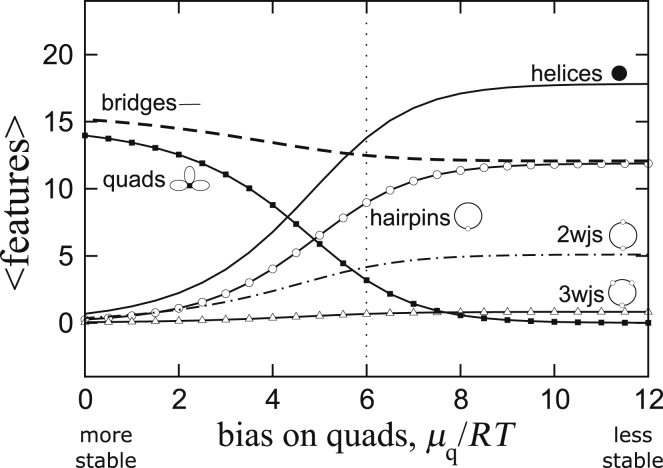
Ensemble averages of the number of helices (*solid line*), bridges (*dashed lines*), hairpin loops (*open circles*), two-way junctions (*dotted dashed lines*), three-way junctions (*open triangles*), and quadruplexes (*squares*) computed from the physically relevant solution for a (CNG)_60_ repeat as a function of quadruplex stability (stable on the *left*, unstable on the *right*).

We have applied the calculations described in Materials and methods to (CNG) repeats, where N = A, C, G, or U, to compute the ensemble average number of secondary structure features associated with the conformations of the chains. The Dyson equation in Fig. 4 is quadratic in *R*_3_, and there are in general two roots. In all of the cases studied, we found only one of them to yield physical results, whereas the other root produced a negative value for the partition function *Z*. Results from the physically relevant solution are shown in Fig. 5. Because (CAG), (CCG), and (CUG) repeat sequences cannot physically produce quadruplexes but (CGG) repeats may, we have plotted the results as a function of the stability of the quadruplex core $\mu_{q}^{0}$/*RT*. Although (CGG) repeat sequences can potentially form quadruplexes, experimental evidence shows little to no quadruplex structures on (CGG)_17_ or (CGG)_20_ sequences (36). On the other hand, (AGG) repeats have been found to fold predominantly into quadruplex-rich structures (36). We have employed experimental data for (AGG) repeats to establish an upper limit for how stable a quadruplex could be if it were to exist in (CGG) repeats. This upper limit is on the left side of the graphs in Fig. 5, and the quadruplex core stability decreases (i.e., $\mu_{q}^{0}$/*RT* becomes more positive) moving to the right. (CAG), (CCG), and (CUG) repeats are therefore associated with the right side of Fig. 5. The expected structural features of (CNG)_60_ chains are displayed as a function of $\mu_{q}^{0}/RT$.

Before discussing the results, we point out that what have been calculated are ensemble averages, and as such, they may contain contributions from a large number of different structures. When considering the data, it is therefore important to not associate the averages with a single conformation, keeping in mind that there may be many structures within each ensemble. For example, although the maximal hairpin structure depicted in Fig. 1 *a* may be one of the prevalent structures in a (CNG) repeat ensemble, it may be only one of many. In fact, the ensembles we have computed are rather diverse, and the averages of all the structural features vary smoothly across the entire parameter space studied.

**Figure 6:**
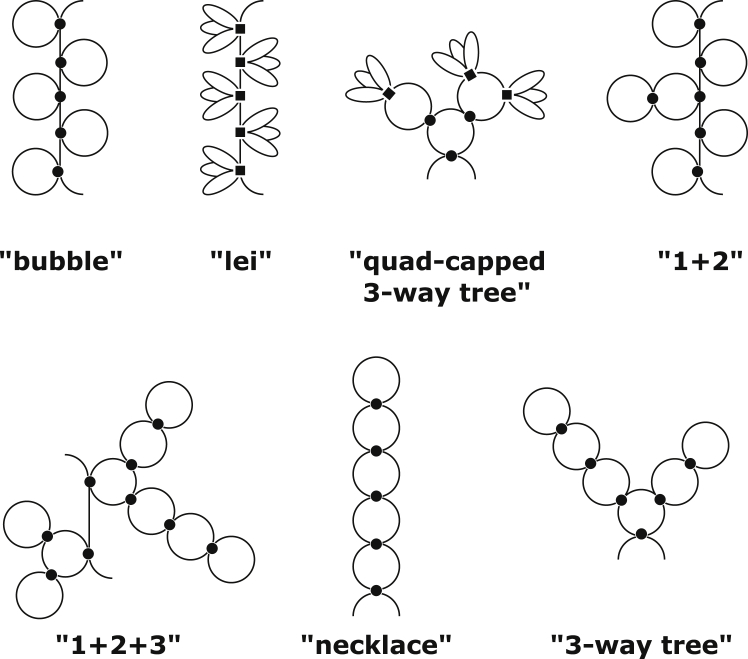
Diagrams illustrating some of the structures observed in the results in Figs. 5 and 7.

Fig. 5 shows that the structural characteristics of (CNG)_60_ are strongly dependent on the ability of the chain to make quadruplexes. When quadruplexes are unstable, the structures on the right side of Fig. 5 correspond to an ensemble with largely open chains with high concentrations of bridges and hairpin loops and some two-way junctions, but relatively few three-way junctions and no quadruplexes. Interestingly, the number of hairpin loops is almost identical to the number of bridges on the right side of Fig. 5. This suggests that the structures in this ensemble are dominated by the “1 + 2” diagrams, an example of which is illustrated in Fig. 6. Furthermore, a large number of bridges is also indicative of largely open structures, but the number of helices observed here is somewhat less than the maximal number that could be sustained on a (CNG)_60_ repeat (the theoretical maximum is 29). Instead of being driven by the favorable enthalpy of formation of the helices, the formations in this ensemble seem to be dominated by loop entropies.

Next, focusing on the left side of Fig. 5, we examine how the presence of quadruplexes alters the structural characteristics of the ensemble. As the stability of the quadruplex is increased (i.e., $\mu_{q}^{0}$/*RT* going from *right* to *left* in Fig. 5), they begin to displace the helices. This is revealed by a decrease in the concentration of helices and a concomitant increase in the concentration of quadruplexes. The number of bridges on the chain also increases, whereas the number of two- and three-way junctions decreases. These changes occur because as the quadruplexes displace the helices, the chain must dissolve other structures to give way to the quadruplexes, as quadruplexes have a larger footprint on the sequence (one quadruplex takes up a minimum of four CNG repeats, whereas a helix only takes up two). Dissolution of the other structures creates more bridge segments. Based on these observations, we can conclude that the most relevant graphs in the stable-quadruplex limit (*left side*) of Fig. 5 are the “lei” diagrams in Fig. 6, where quadruplexes are distributed along a largely open chain.

Experimental evidence shows little to no quadruplex formation for short (CGG) repeat sequences (36). Based on this and the results in Fig. 5, we can estimate that the stability of a quadruplex on a (CGG) chain $\mu_{q}^{0}$/*RT* must be at least ∼6*RT* lower than on an (AGG) chain. We indicate this estimate in Fig. 5 by a vertical dotted line. This suggests that a quadruplex in (CGG) repeats must be approximately >3.7 kcal/mol less stable than in (AGG) repeats.

**Figure 7:**
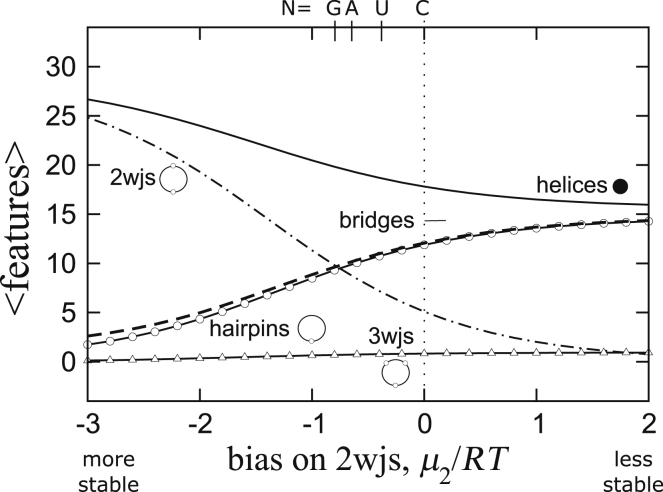
Ensemble averages of features computed for a (CNG)_60_ repeat as a function of extra stability added to each two-way junction (favorable on the *left*, unfavorable on the *right*).

Next, we examine the structures of (CNG) repeat in the absence of quadruplexes. As we have seen already, even though (CGG) repeats can form quadruplexes, quadruplexes in (CGG) repeat are expected to be ∼3.7 kcal/mol less stable than those in (AGG) repeats. The other (CNG) repeats, N = A, C, and U, cannot physically form quadruplexes. In Fig. 7, we show results for these (CNG) repeats after placing a large unfavorable bias against quadruplex formation on the chains.

In actual (CNG) repeat sequences, the ability of the N nucleotides to form noncanonical basepairs is expected to favor different structures depending on whether N = A, C, G, or U. We can capture these effects in our model by assigning an extra bias to the two-way junctions of those sequences in which a noncanonical basepair or stacking can add stability to the chain. The bias is applied to every two-way junction regardless of size primarily to account for the propensity of stacking a N nucleotide against either of the helices on the junction. To easily ascertain these effects, the results in Fig. 7 are reported as a function of this bias *μ*_2_/*RT*, where *μ*_2_ is a chemical potential imposed on each two-way junction. Negative value adds a bonus, and positive value assesses a penalty. Approximate values of the bias for N = G, A, U, and C are indicated on the top of Fig. 7.

In the limit at which two-way junctions are very stable (*left side* of Fig. 7), the structures are dominated by a large number of helices and two-way junctions but very few hairpins or bridges. This suggests that the ensemble is characterized by closed and compact structures. These conformations correspond to the “necklace” diagrams in Fig. 6 that we have discussed in Materials and methods.

Turning to the right side of Fig. 7, in the limit of a large bias imposed against the formation of two-way junctions, the solutions correspond to the “bubble” diagrams in Fig. 6, and they are the hairpin-capped counterpart of the lei diagrams. They have almost as many bridge segments as hairpins, but the number of helices is far from the theoretical maximum of 30. These chains are therefore largely open, and they are dominated by the entropies of the loop segments. Results from Fig. 7 suggest that noncanonical basepairs or favorable stacking of the N nucleotide within the junctions can produce a significant effect on the conformations of (CNG) repeats. The values of the bias *μ*_2_/*RT* used to generate the results in Fig. 7 span a range of only ∼3.1 kcal/mol, but within this very narrow range, the structures in these ensembles vary drastically.

**Figure 8:**
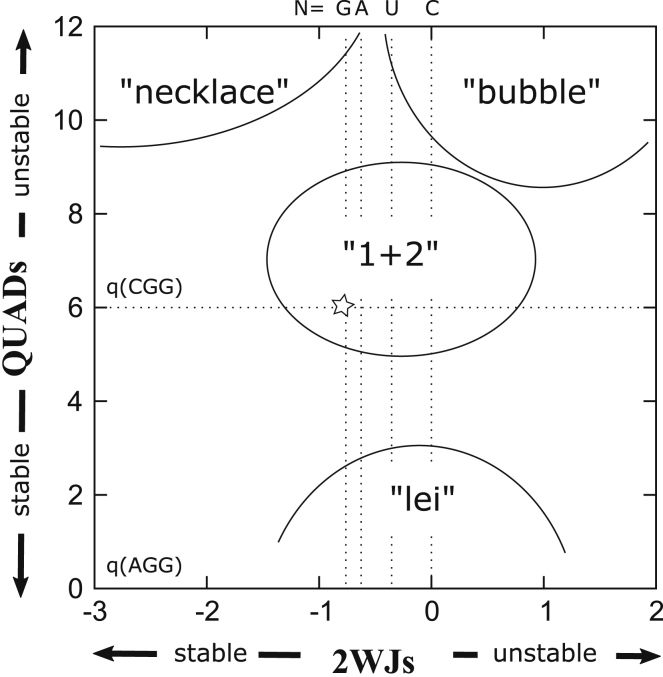
A “phase diagram” summarizing the results from Fig. 5 and 7. The horizontal axis indicates two-way junction stability, and the vertical axis quadruplex stability. Phases that have been identified by the calculations are labeled. See Fig. 6 for their graphical representations. Phase boundaries are approximate. Star shows position for which the scaling analysis in Fig. 9 was carried out.

Fig. 8 shows a “phase diagram” summarizing all the findings from above, in which variations in quadruplex stability from Fig. 5 are plotted along the vertical direction and variations in two-way junction stability from Fig. 7 are plotted along the horizontal direction. On this phase diagram, “(AGG)” and “(CGG)” indicate the approximate quadruplex stabilities in (AGG) versus (CGG) chains. Approximate values of the stability of two-way junctions in (CNG) repeats for N = G, A, U, and C are also indicated on the top of Fig. 8. Non-quadruplex-forming (CNG)_60_ repeat sequences occupy the center of this phase diagram, with most of their structures dominated by the 1 + 2 and bubble diagrams illustrated in Fig. 6, which are semiopen structures. A minor fraction of the ensemble is also made up of necklace structures, which are closed and compact. These results point to the existence of many potential structures of similar prevalence with contributions from both open and compact structures. Though the crystallographic data of (CNG) repeats suggest the dominance of hairpin structures (32,38,41), this leaves open the question of how an ensemble of diverse structures could be detected in solution. Techniques such as small-angle x-ray scattering (60, 61, 62, 63), ultraviolet melting (64), and Förster resonance energy transfer (65) can all be used to probe the solution structure of RNA. Although the use of thermodynamic data of Sobczak et al. (36) does provide a point of contact between the calculated free energies and experimental measurements, the ensemble predicted by our results is diverse enough that a one-to-one correspondence to specific structure(s) revealed by experiments is unlikely. Also important is that multiway junctions seem to be of low abundance because higher branching costs more entropy according to the data in Table 1, so although multibranch structures higher than three-way can be included in the calculations, they are not likely to alter the results significantly.

**Figure 9:**
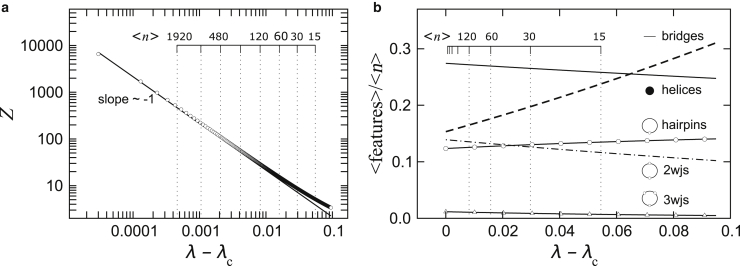
(*a*) Divergence of the partition function *Z*(*λ*) when *λ* approaches the singular point *λ*_*c*_. The scale on the top shows the average repeat lengths 〈n〉 for each *λ*. (*b*) Structural features as a fraction of the repeat length as a function of *λ*. The scale on the top maps 〈n〉 to *λ*. Short repeats and long repeats have very different structural compositions, and the crossover appears to occur between 30 and 60 repeats.

The conformational ensembles are functions of the repeat length. This repeat length dependence is illustrated in Fig. 9 for a point on the phase diagram marked by the star in Fig. 8. Fig. 9 *a* shows divergence of the partition function *Z*(*λ*) when *λ* approaches the singular point *λ*_*c*_. The slope is ∼−1, suggesting that it is a simple pole. This result is expected because this problem is isomorphic to the enumeration of all paths from the 5′ to 3′ end of the chain on the space the folding problem is embedded in, and the generating functions of paths all have the same dominant singularity, which is a simple pole (66). The scale on the top of Fig. 9 *a* shows the average repeat lengths 〈n〉 for each *λ*, and repeat lengths approximately >60 appear to be in the scaling region. Fig. 9 *b* shows how each of the features as a fraction of the repeat length varies as a function of *λ*, again with the scale on the top mapping 〈n〉 to *λ*. Short repeats and long repeats have very different structural compositions, and the crossover appears to occur between 30 and 60 repeats. Note that in the scaling limit, there are almost equal densities of bridges, hairpins, and two-way junctions on the chain, and the ensemble is dominated by largely open structures.

**Figure 10:**
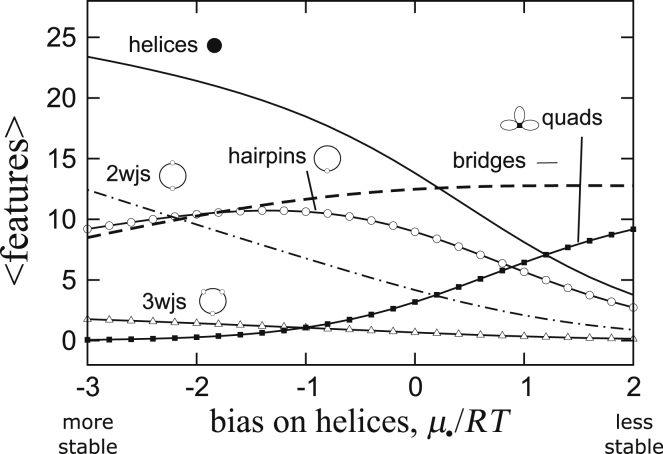
Ensemble averages of features computed for a (CNG)_60_ repeat as a function of extra stability added to each helix (favorable on the *left*, unfavorable on the *right*).

Finally, because there is a significant discrepancy between the stability of the GC|CG duplexes predicted by NNDB compared to experimentally derived results collected specifically from (CNG) repeat sequences, we want to know to what extent the stability of the duplexes may have an effect on the computed results. Fig. 10 shows the structural characteristics of (CNG)_60_ as a function of a bias placed on the helices, with more stable to the left and less stable to the right. Toward the right, as the helices become less stable, they are displaced by quadruplexes, which are the only structures other than helices that can cap the end of a branch. These map to the lei diagrams in Fig. 6. Toward the left, as the helices become more stable, they seed an increasing number of two- and three-way junctions in favor of hairpins. The resulting structures correspond to the necklace and “three-way tree” structures in Fig. 6. Notice that −3*RT* on the left edge of Fig. 10 corresponds to only −1.8 kcal/mol of extra stability, and this small difference produces a significant change in structural compositions. Therefore, a more accurate experimental assessment of the thermodynamic stability of the GC|CG duplexes may be important for understanding (CNG) repeat structures.

## Conclusions

We have formulated a diagrammatic theory to study the conformational ensembles of (CNG)_n_ RNA sequences. Transcripts of overexpanded microsatellites on the genome containing 60–100 (CNG) repeats have been implicated in a number of neurological diseases known as TREDs. To understand the structures of these (CNG) repeat sequences, we performed a series of calculations aimed at characterizing their equilibrium ensembles. With a diagrammatic representation of the partition function, our calculations are based on using graphs to annotate structural motifs on the chains, and, in conjunction with evidence from previous simulation studies, these diagrammatic representations allowed us to easily factorize the graphs to re-express the free energy of each configuration as a sum of independent terms. Using generating function mathematics and diagrammatic re-summation techniques, we were able to derive a closed-form expression for the partition function in terms of a renormalized root function, which is the diagrammatic equivalence of the sum over all self-contained circuit diagrams. Employing a simple approximation for this root function, we derived analytical expressions for the partition function and its corresponding thermodynamic observables. Including hairpins, two- and three-way junctions, helices, and quadruplexes in the root function, the partition function captures an infinite set of conformations with any number and any combination of these structural elements. Together with simulation data from a self-consistent library of entropic costs previously obtained for the various graph elements, as well as experimentally derived free energies for the helices and quadruplexes, we solved the resulting equations to arrive at numerical estimates for the ensemble expectation values of the number of structural features on the chain, including bridges; hairpin loops; one-, two-, and three-way junctions; and quadruplexes. This enabled us to quantitatively characterize the structural diversity of different (CNG)_n_ ensembles.

Whereas most studies in the field have implicitly assumed that the ensemble of a (CNG)_n_ sequence is dominated by a single structure having the maximal number of paired bases forming duplexes interposed by two-way junctions between them, the results of this study suggest otherwise (27,35,36,38,39). The data show that the structural ensembles of (CNG)_n_ repeat sequence with n ∼60 are surprisingly diverse. The equilibrium number of duplexes, hairpins, junctions, bridges, and quadruplexes on these sequences indicate that their secondary structure contents are far from the expected maximally paired conformation. To the contrary, the ensemble is dominated by a mixture of open and compact structures. We have mapped out the resulting structures as a function of the ability of the N nucleotide (N = A, C, G, or U) in (CNG) repeats to make noncanonical pairs, as well as their ability to sustain stable quadruplexes. The “phase diagram” that emerges shows a diversity of different structures across this parameter space, demonstrating that ensembles of (CNG) repeat sequences can potentially contain many alternate conformations. The results show how perturbations in the form of biases on the stabilities of the various structural motifs—duplexes, junctions, hairpins, and quadruplexes—could affect the secondary structures of the chains in either directions and how these structures may switch when they are perturbed, e.g., when they interact with or bind other molecules. This may, in turn, have implications on how these (CNG)_n_ sequences could acquire unintended functions in the cell, leading to their cytotoxicity.

## Author contributions

C.H.M. designed the study. C.H.M. and E.N.H.P. carried out the work. C.H.M. and E.N.H.P. wrote the manuscript.
